# Exclosure Enhances Crop Yields and Rural Livelihood Resilience in Northern Ethiopia

**DOI:** 10.1007/s00267-026-02413-4

**Published:** 2026-03-31

**Authors:** Gidey Kidu Mezgebo, Edward Lahiff, Tracy Bradfield, Stephen Thornhill

**Affiliations:** 1 University College Cork, Cork City, Ireland; 2 Mekelle University, Mekelle, Ethiopia

**Keywords:** Dryland restoration, Household survey, Livelihood resilience, Exclosure, Tigray

## Abstract

Ethiopia’s food production and rural livelihoods are increasingly threatened by land degradation and climate variability, particularly in dryland areas vulnerable to climatic shocks. Exclosure, a designated area shielded from human and animal disruption, has emerged as a vital restoration approach, especially in northern Ethiopia. Empirical studies have largely focused on ecological outcomes and have provided limited and mixed results on whether exclosure enhances agricultural productivity and livelihood diversification. This study addresses this gap by examining the effect of exclosure on crop yields and household livelihood diversification using panel data from 491 households gathered over two consecutive cropping years, across varying climate conditions. Fractional logit model, propensity score matching, and Hausman-Taylor estimations were employed to compare households living downstream of the exclosure with those in open grazing areas. The results indicate that households downstream of exclosures exhibit markedly greater livelihood diversification than those with open grazing areas, and this is influenced by age, gender, livestock ownership, extension services, financial availability, and access to marketing information. The analysis also reveals a positive effect of exclosure on crop yield per hectare, with more pronounced differences observed during drought conditions. Overall, the findings demonstrate that exclosure contributes not only to ecological restoration but also to stabilizing agricultural production, strengthening food security, and enhancing rural livelihoods resilience. The study provides empirical evidence to inform national and regional policy makers, practitioners, and land management planners of the benefits of exclosure as a restoration approach and supports their integration and scaling up in degraded and climate sensitive regions.

## Introduction

Land degradation remains a major challenge across Sub-Saharan Africa (SSA), with varying degrees of severity across landscapes and ecosystems (Lakew Tefera et al. 2024; Nkonya et al. 2016). In East Africa, about 40% of soil is degraded, undermining agricultural productivity, worsening poverty, and exacerbating food insecurity (Lakew Tefera et al. 2024; Okello et al. 2021). When compounded by climate change and conflict, land degradation intensifies soil erosion and run off and poses a serious obstacle to achieving the Sustainable Development Goals (SDGs) (Imasiku et al. 2020; Mirzabaev et al. 2023). Drivers of degradation include rapid urbanization, unsustainable farming practices, overgrazing, deforestation, and rising energy demand, together with climate shocks and conflict (Slayi et al. 2024). These pressures have contributed to widespread poverty (Mesele et al. 2025), with 52% of SSA’s population experiencing multidimensional poverty and Ethiopia’s rate reaching about 69% (UNDP and OPHI, 2024).

Agriculture is central to rural livelihoods and economic growth in SSA, supporting food security and providing various inputs for industry (Amede et al. 2023; Neglo et al. 2021). Yet, resource degradation, limited livelihood, fragmented landholdings, fragile institutional support systems, and weak integration of resource conservation into production system constrain the sector (Atukunda et al. 2021; Viana et al. 2022). Under conditions of degraded land, recurrent drought, and small plots, diversification and productivity gains are essential strategies for smallholder resilience (Ellis 1998).

In Ethiopia, Agriculture supports more than three-quarters of the population, but is increasingly threatened by climate variability, conflict, and natural resource degradation (Abdi et al. 2023; Neglo et al. 2021; Yigezu Wendimu, 2021). These problems are particularly acute in northern Ethiopia, especially Tigray, where civil unrest and severe land degradation have compounded household vulnerability (Tessema, 2024; Wassie, 2020; Yigezu Wendimu, 2021). Most smallholders operate at near-subsistence level, with limited access to markets and persistent food insecurity (Seyoum, 2024; Solomon et al. 2024). Enhancing crop productivity and livelihood diversification is therefore crucial for building resilience: higher yields generate income for diversification, while diversified livelihoods facilitate investment in modern inputs such as fertilizers and improved seeds (Bamidele, 2024; Tasnim et al. 2025).

To address land degradation, Ethiopian farmers have executed numerous land management strategies, including agroforestry, reforestation, soil bunds, intercropping, and the adoption of drought-resistant crops (Rapiya et al. 2024; Solomon et al. 2024; Teku and Derbib 2024; Tessema, 2024). Exclosure, a designated area excluded from human and livestock disturbances to facilitate natural regeneration, has been extensively implemented in northern Ethiopia, especially in Tigray, and across East Africa (Adem et al. 2020a, 2020b, 2024; Mekuria et al. 2021; Tucker and Murphy 1997). Exclosure is established to restore degraded land by enhancing natural capital through improving vegetation cover, soil fertility, water retention, and biodiversity, particularly on steep erosion-prone slopes in dryland areas Gebremedhin et al. (2003), Dangiso and Wolka (2023), Araya et al. (2023) and Ereso (2024), Conversely, open grazing lands often experience continued degradation, limiting their capacity to support productive and diverse livelihoods (Cao et al. 2019; Tessema, 2024).

Recent studies from the agronomic aspect noted that land restoration interventions such as soil and water conservation, integrated soil fertility management conservation agriculture and agroforestry have a positive impact on agricultural productivity Malan et al. (2024),. Similarly, exclosure as land restoration intervention has widely documented to deliver important ecological and biophysical benefits, including soil restoration, biodiversity recovery, and hydrological enhancements, especially in Ethiopia and more generally in East Africa and in other countries such as China (Araya et al, 2023; Cao et al. 2019; Solomon et al. 2024). Nonetheless, evidence regarding their socio-economic, particularly its impact on crop yield and livelihood diversification remains limited. Prior research has predominantly been descriptive, concentrating on the perceived accessibility of resources such as fuelwood, fodder, and thatching grass (Atsbha et al. 2025; Mezgebo et al. 2022). Only a limited number of thorough analyses are available. Mekonen (2018) analyzed how the outputs of exclosure are distributed and the factors that influence the distribution, perception and attitudes of the community, while Araya et al. (2023a) assessed income contributions from exclosures. Nevertheless, neither study assessed the impacts of the exclosure on livelihood diversification and crop productivity. Furthermore, as noted by Zheng et al. (2018), the net advantages of restored lands across various scales are still debated.

The research gap is particularly notable in Tigray, where exclosure has been extensively utilized for numerous years, yet the effect on household economic resilience remains largely unexamined. Accordingly, this study tests the following hypotheses: (H1) households located downstream of an exclosure achieve significantly higher crop yields per hectare than households in open grazing areas; and (H2) households located downstream of an exclosure have greater livelihood diversity in comparison to households with unrestricted grazing land. We employed empirical data collected over two production years from households located downstream of the exclosure and those on open grazing land. This study makes a novel contribution by providing panel data on the combined effect of the exclosure on crop productivity and household livelihood diversification, an aspect that has received limited attention in prior research, particularly under varying climate conditions. It also offers novel insights for policymakers, practitioners, and community stakeholders into how the restoration of degraded land through exclosure, initially intended to promote ecological recovery, links with sustainable economic development.

## Theoretical Framework

This study utilises Scoones’ (1998) Sustainable Livelihood Framework (SLF) to investigate how land restoration through exclosure affects livelihood diversification and crop productivity in rural Tigray, Ethiopia. The SLF suggests that sustainable livelihoods depend on the interaction of five forms of capital: natural, human, social, physical, and financial.

Ecological improvements from exclosure (*natural capital*) are expected to generate spillover effects across other livelihood capitals. Enhanced soil fertility, forest recovery, stabilized microclimate, and hydrological cycles can strengthen *human capital* by supporting new agricultural practice such as cut‑and‑carry forage feeding and soil and water conservation (Teku et al. 2025). Furthermore, collective management of exclosure resources, such as fodder and fuelwood, builds *social capital* by promoting cooperation and shared responsibility within communities (Araya et al. 2023b; Gebregziabher and Soltani 2019). Exclosure also improves *physical capital* by increasing water retention (by 15–25%) and reviving lost spring, supporting irrigation and beekeeping activities (Teku et al. 2025; Atsbha et al. 2025). Increased access to fodder, fuelwood, and bee flora are also presumed to open additional income‑generating opportunities, enhancing the *financial capital* of farmers and supporting more diverse livelihoods (Kibret et al. 2022; Mezgebo et al. 2022).

However, outcomes are shaped by contextual factors such as demographic characteristics, socioeconomic status, institutional access, and environmental conditions. While exclosure may involve trade-off, including opportunity costs and uneven benefit distribution, these issues lie beyond the scope of this quantitative analysis. Guided by the SLF, our study hypothesizes that households living downstream of exclosures have (i) higher livelihood diversification and (ii) greater crop yields than households in comparable open grazing areas, reflecting differences in the forms of capital.

## Methodology

### Description of the Study Area

**Fig. 1:**
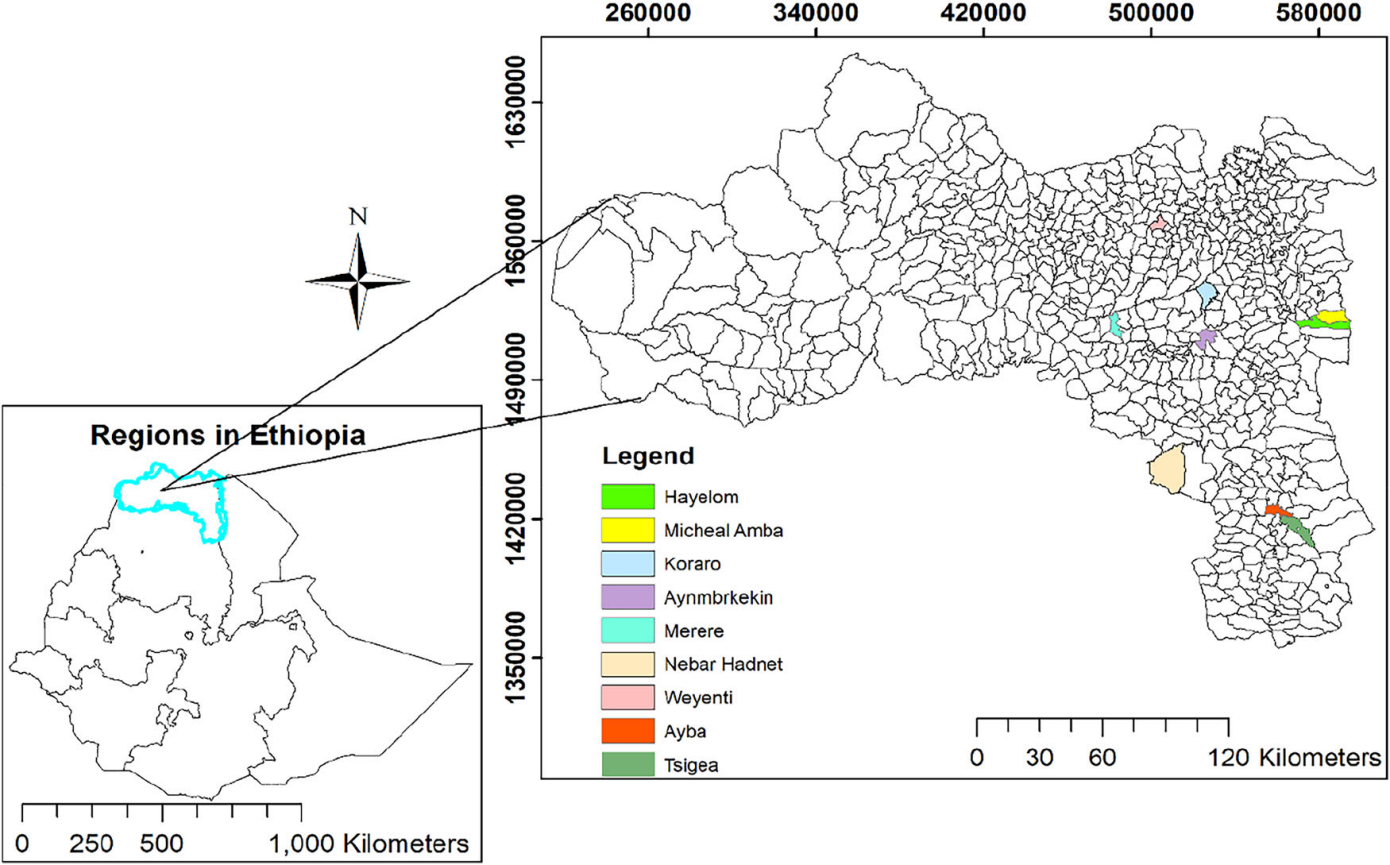
Map of the study sites

The study was conducted in the Tigray regional state in northern Ethiopia (Fig. 1), bordering Eritrea, Sudan, Afar, and Amhara (Yoahannes, 2021). The region features diverse topography ranging from highland plateaus to rugged maintains to eastern lowland drains and is drained by major rivers such as Tekeze and Mereb.

The region has a predominantly semi-arid climate, with a short rainy season (June–September) and a long dry season (October–May). Mean annual rainfall in the study areas is about 587 mm (National Meteorological Agency (NMA) of Ethiopia, 2015), contributing to chronic soil erosion and water scarcity that constrain agricultural production, the main livelihood. Land use is dominated by cropland (40%), shrubland (28.8%), and forest (13.4%), while wetlands and water bodies together account for less than 1% (Ethiopian Environmental Protection Authority (EEPA), 2025), with sever land degradation driven by overgrazing and climate variability. To counter these impacts, soil and water conservation measures were introduced in the 1970s, particularly terracing and stone bunds in the highlands (Gebremedhin and Gebremicael, 2018; Munro et al. 2019). The implementation of exclosure started in the 1980s, through joint efforts of local communities and government institutions, becoming an integral component of land rehabilitation strategies (Birhane et al. 2017; Naudtsayb et al. 2004). This study covers four administrative Zones (Zoba^1^) (central, eastern, southeastern, and southern Tigray), eight districts (Woreda^2^) and nine kebeles or *Tabia*^3^, selected to represent three agroecological zones differing in altitude.

## Sampling Procedure

We used a multi-stage sampling process to select study sites and households. In the first stage, four of the seven administrative Zones in Tigray were chosen based on accessibility and the presence of exclosures. The western and northwestern Zones were excluded due to insecurity after the 2020–2022 conflict, and Mekelle city was excluded because it lacks exclosures. Second, eight districts (Woreda) were randomly selected from the four Zones and grouped by agroecology: three highlands, two midlands, and three lowlands. In the third stage, within each agroecological zone, three exclosures of different ages (young, medium, and old) were purposively selected from these districts, totaling nine exclosures. In the lowlands, Tsada Emni, Tunseka, and Chilkomisreta represented the three exclosure age categories; in the midlands, Merere, Abel Degaw, and Seyabo; and in the highlands, Zban Brle, Maybaeti, and Arera (Table 1). Fourth, two villages were purposively selected from each of the nine kebeles: one located downstream of the exclosure (by age category) and another based on open grazing, resulting in 18 villages overall. Household lists were obtained from the local Office of Agricultural and Natural Resources. The total sample size, determined using Yemane’s (1987) formula, was 514 households^4^, chosen through systematic random sampling with proportional allocation across villages^5^. Ex-post statistical power analysis confirms that the sample size is sufficient^6^.

**Table 1:** Name of selected exclosure by age, agroecology, number of respondents, etc

Name	Age	Agroecology	Exclosure group	Control group	Tabia/kebelle	District
*Arera*	33	Highland	32	31	*Ayba*	Alaje
*Maybeati*	22	Highland	26	27	*Aynbrkeken*	Degua tembien
*Zban Birle*	17	Highland	19	20	*Micheal Emba*	Tsirea wenberta
*Seyabo*	33	Midland	18	19	*Weyenti*	Adwa
*Abel dega*	22	Midland	38	38	*Hayelom*	Tsirea wenberta
*merere*	17	Midland	26	27	*Merere*	Kola tembien
*Chilko misreta*	33	Lowland	36	28	*Tsigea*	Raya azebo
*Tseda Emni*	22	Lowland	24	24	*Nebar Hadnet*	samre
*Tunseka*	17	Lowland	29	29	*Koraro*	Hawzien
Total			248	243		

## Data Collection Procedure

Primary data were collected through face-to-face household surveys with households downstream of exclosures and open grazing lands. A survey was conducted in two rounds to capture the 2023 (2015/16 Ethiopian Calendar) and 2024 production years (2016/17 Ethiopian Calendar). The questionnaire, implemented in the local Tigrinya language, captured self-reported crop production, demographics and socioeconomic characteristics, input use, consumption, and livelihood activities. The questionnaire was pretested with 30 households and refined accordingly, and enumerators received training on survey administration, ethical protocols, and data collection.

## Operational Variables

This section presents the operational definitions of all the variables used in the analysis. Each is described in terms of its definition, method of measurement, and expected impact on the outcome variables (livelihood diversification and crop yield). Table 2.

**Table 2:** Variable definitions, measurement, and expected effects

Variables	Description & measurement
Age	Age of the household head, in years. Older heads are expected to have more experience, which may enhance livelihood diversification and crop productivity.
Gender	Dummy variable: 1 = male, 0 = female. Gender may influence access to resources and decision-making power, which can affect productivity and diversification.
Marital Status	Categorical variable: 1 = single, 2 = married, 3 = divorced, 4 = widowed. Marital status may affect resource access, labor, and stability, influencing outcomes in either direction.
Education	Formal schooling of the household head in years. More education is hypothesized to broaden livelihood options and enhance productivity.
Family size	Number of individuals in the household, of any age. Larger families provide more labor and can pursue varied strategies, potentially boosting productivity and diversification.
Distance to the market	Minutes required to walk to market (self-reported). Greater travel time is expected to hinder access to markets, reducing opportunities and productivity.
Distance to farmers’ training center (FTC)	Walking minutes to nearest FTC (self-reported). Greater proximity (fewer minutes) improves access to information and support, likely enhancing outcomes.
Membership in local organization	Dummy: 1 = member of any farmers’, savings, or youth association; 0 = not a member. Membership facilitates access to resources and information, expected to have positive effects.
Soil conservation on farmland	Dummy: 1 = practices soil conservation; 0 = does not. Soil conservation can improve productivity but may initially divert resources, yielding mixed effects.
Access to credit	Dummy: 1 = has received credit; 0 = has not. Access to financial resources can enhance investment and productivity, though effects may vary by context.
Access to extension service	Dummy: 1 = receives support; 0 = does not. Extension services provide valuable knowledge, usually increasing productivity and diversification.
Access to marketing information	Dummy: 1 = has access; 0 = does not. Access to timely market information is expected to broaden options and improve returns.
Soil fertility	Categorical: 1 = low fertility, 2 = medium, 3 = high (as perceived by household). Higher fertility is expected to improve productivity.
Plot slope	Categorical: 1 = flat, 2 = medium, 3 = steep. Steeper slopes may reduce productivity and restrict livelihood options.
Fertilizer	Total kg applied to fields. More fertilizer use typically boosts yields per hectare.
Labor per hectare	Calculated as total person-days of labor divided by land under cultivation. More labor generally raises productivity.
Livestock holding	The number of Tropical Livestock Units used by the household. More livestock can increase household income, food security, and the supply of organic fertilizer.

## **Method of Data Analysis**

Survey data were cleaned and coded. Descriptive statistics and independent t-tests were used to compare livelihood outcomes between households downstream of the exclosure and those with open grazing lands. To examine determinants of livelihood diversification and estimate the impact of the exclosure on crop productivity, we employed a combination of fractional logit model, propensity score matching (PSM) with entropy balancing, and panel data estimation using Hausman-Taylor (HT) model.

## **Analytical Framework and Model Justifications**

This study integrates descriptive analysis, cross-sectional causal inference and panel estimation to evaluate the effect of exclosure-based land restoration while addressing selection bias, unobserved heterogeneity and the bounded nature of key outcome variables. Livelihood diversification is measured using Simpson diversity index (SDI) and its determinants are analyzed using a fractional logit model, which is appropriate for proportional outcomes bounded between 0 and 1. Because households are not randomly assigned to live downstream of exclosure, propensity score matching (PSM) with entropy balancing is used to construct comparable treatment and control groups and estimate treatment effects on crop yield. Finally, the panel structure of the data is exploited using the Hausman Taylor estimator to control for time invariant heterogeneity and potential endogeneity while retaining time invariant regressors such as land management and agroecological zone.

## **Livelihood Diversification and Model Specification**

Livelihood diversification index (LDI) was measured using the SDI method, which captures both the number of income sources and the evenness of income distribution, and is preferred over Shannon-Weiner Index or simple counts of income sources (Alemu, 2023; Roscher et al. 2022). The SDI ranges from 0 (only single source or complete specialization) to 1 (highest diversification) and is computed as shown in Eq. (1).

1 $${SDI}=1-\mathop{\sum }\limits_{i=1}^{n}{\rho }_{i}^{2}$$

where n is the number of income sources and ρ is the proportion of income from, i source.

To analyze factors associated with diversification, we employed the fractional logit model which accommodates the bounded nature of the SDI and allows for observation at 0 and 1 without transformation and is preferred over the Beta regression, which requires observations strictly within the (0,1). It is more suited for proportional data, does not require normality or homoskedasticity and ensures predicted values remain within the logit link (Ojo et al. 2024, Swathi. M and Sridharan 2022). The model is specified as follows:

2 $$E\left(\frac{{SDI}}{X}\right)=\frac{1}{1+\exp (-X\beta )}$$

SDI measures a household’s livelihood diversification, bounded between 0 and 1. X is a vector of explanatory variables, and β denotes estimated coefficients.

## **Crop Yield and Model Specification**

This study focused on crop productivity to assess the agricultural output of exclosure, excluding livestock. This is because livestock ownership in Ethiopia is primarily intended to support crop production and provide draft power. It is also widely held as a store of wealth or insurance rather than for short term productive use, making changes over two-year period an unreliable indicator of productivity (FAO 2018).

Since decisions to close open grazing lands are made collectively by officials and community leaders (Gebregziabher and Soltani 2019b), households downstream of the exclosure are not randomly assigned. To address this potential bias, we used Propensity Score Matching (PSM) combined with Entropy Balancing for each survey year (Zewdie et al. 2025). Propensity scores were estimated using a logit model based on household demographics, plot characteristics, agroecology, institutional access, livestock holding and location variable (Eq. 3). Matching was performed using nearest-neighbor, radius and kernel algorithms to construct comparable groups. Post-matching diagnostics, such as standardized mean differences (SMD) and common support checks, confirmed the adequacy of the matching procedure. The average treatment effect (ATE), average treatment effect on the treated (ATT), and average treatment effect on the untreated (ATU) to assess crop productivity outcomes were estimated using Eqs. (4) and (5). ATT was derived directly from matched samples, while ATE and ATU were estimated by averaging over treated, control, and full populations, respectively, using both PSM and entropy balancing methods (Tübbicke, 2022).

3 $$P\left({D}_{i}=1|X={X}_{i}\right)=P\left({X}_{i}\right)$$

where ${D}_{i}$ is the dependent (treatment) variable, taking the value of 1 if the household lives downstream of the exclosure and 0 otherwise. ${X}_{i}$ is a vector of covariates. $P({X}_{i})$ is the propensity score to be estimated, representing the probability that a household lives downstream to an exclosure or with open grazing land, given the observed covariates.

4 $${ATE}=E[{Y}_{1}-{Y}_{0}]$$

where Y_1_ and Y_0_ are the crop productivity of households located downstream of the exclosure and open grazing land, respectively.

5 $${ATT}=\frac{1}{{N}_{T}}\mathop{\sum }\limits_{i:{D}_{i}=1}({Y}_{i}-{Y}_{j(i)})$$

where the ${N}_{T}$ is the number of households living adjacent to exclosures $({D}_{i}=1$), ${Y}_{i}$ represents the observed crop yield per hectare for household i living downstream of an exclosure, and, ${Y}_{j(i)}$ is the counterfactual crop yield per hectare for the same household if it had lived in open grazing land (estimated using matched controls). Entropy balancing provides weights that ensure covariate distributions in the control group mirror those in the treated group, allowing for doubly robust, population-level inference.

To analyze crop productivity over time, and to account for unobserved heterogeneity and potential endogeneity, we employed the Hausman–Taylor (HT) model, developed by Hausman et al. (1981) and extended by Baltagi (2023). The HT estimator is well-suited for panel data that includes both time-invariant regressors (e.g., land management type, agroecological zone, household characteristics) and time-varying regressors (e.g., crop inputs). Unlike fixed-effects models, which exclude time-invariant regressors, and random-effects models, which assume full exogeneity, the HT model allows some regressors to be endogenous and uses internal instruments from the panel structure for consistent estimation (Amoroso et al. 2022; Ao, 2009; Baltagi, 2023). The model specification is presented in Eq. (6).

6 $${Y}_{{it}}={\beta }_{0}+{\beta }_{1}{X}_{1{it}}+{\beta }_{2}{X}_{2{it}}+{\gamma }_{1}{Z}_{{it}}+{\gamma }_{2}{Z}_{2{it}}+{\mu }_{i}+{\varepsilon }_{{it}}$$

where.${y}_{{it}}$ is crop productivity for household i in year t ${X}_{1{it}}$ and ${X}_{2{it}}$ are vectors of time-varying exogenous and endogenous regressors, respectively; ${Z}_{1{it}}$ and ${Z}_{2{it}}$ are vectors of time-invariant exogenous and endogenous regressors, respectively.${u}_{i}$ is the panel-specific random effect and ${\varepsilon }_{{it}}$ is the idiosyncratic error term.

## **Results**

### **Demographic Characteristics of Respondents**

Table 3 summarizes the demographic and socioeconomic characteristics of the sample. Most household heads were male (77%) and married (84%), with an average household size of six members and low educational attainment about three years of formal schooling. No statistically significant difference was observed between exclosure and open grazing households in terms of gender, marital status, household size, or years of schooling.

**Table 3:** Descriptive statistics of variables (*N* = 514)

Variable	Exclosure		Open Grazing Land		P value /ch2
	Mean	S.D.^a^	Mean	S.D.	
Labor per hectare	69.44	54.99	61.61	47.48	0.08
Total fertilizer (kg)	3.94	8.16	2.47	4.86	0.01
Age of the respondents (years)	49.30	13.21	48.61	13.06	0.55
Education in years	2.84	3.50	2.97	3.52	0.69
Family size of the household	6.00	2.06	6.17	2.12	0.35
Distance to market (walking minutes)	59.36	52.50	65.24	54.72	0.21
Distance to farmer training center (walking minutes)	33.21	26.37	34.84	24.34	0.47
Tropical livestock unit (TLU)	3.88	2.31	3.56	1.80	0.10
Categorical variable	Freq	%	Freq	%	
Soil fertility (Low)	84.00	16.34	75.00	14.59	0.20
Soil fertility (Medium)	122.00	3.73	132.00	25.68	
Soil fertility High	59.00	11.48	42.00	8.17	
Plot slope (flat)	162.00	31.52	158.00	30.74	0.44
Plot slope (medium)	64.00	12.45	49.00	9.53	
Plot slope (steep)	39.00	7.59	42.00	8.17	
Agroecology (lowland)	79.00	15.37	78.00	15.18	0.75
Agroecology (midland)	87.00	16.93	86.00	16.73	
Agroecology (highland)	99.00	19.26	85.00	16.53	
Gender (female)	61.00	11.88	57.00	11.09	0.97
Gender (male)	204.00	39.69	192.00	37.35	
Soil conservation on farmland (yes)	203.00	39.49	186.00	36.19	0.62
Soil conservation on farmland (no)	62.00	12.06	63.00	12.26	
Membership of local organization (yes)	132.00	25.68	105.00	20.43	0.08
Membership of local organization(no)	133.00	25.88	144.00	28.02	
Access to credit (yes)	98	19.96	91	18.53	0.64
Access to credit (no)	150	30.55	152	30.06	
Access to extension service (yes)	153	31.16	128	26.07	0.04
Access to extension service (no)	95	19.35	115	23.42	
Access to marketing information (yes)	136	27.70	129	26.27	0.70
Access to marketing information (no)	112	22.81	114	23.22	

^a^Standard deviation

Regarding crop production, both groups relied on labor, seed, and fertilizer inputs. Labor use was slightly higher among exclosure households, although not statistically significant (*p* = 0.08). Fertilizer application was significantly greater in exclosure areas (3.9 kg/ha vs. 2.5 kg/ha; *p* = 0.01). Environmental and institutional characteristics were largely comparable between groups. The only significant institutional difference was access to extension services, which was higher among exclosure households (31.2% vs. 26.1%; *p* = 0.04). Overall, the two groups were broadly comparable, with differences mainly in fertilizer and extension service.

## Exclosure and Livelihood Diversification

**Fig. 2:**
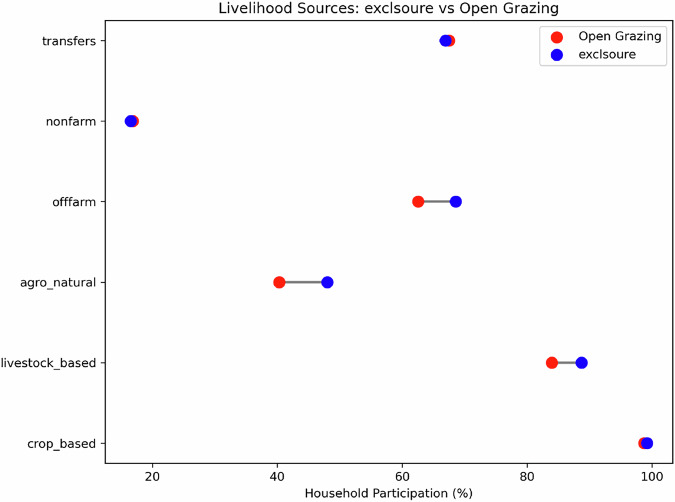
Livelihood sources in the study sites. Note; transfer refers to income from government transfer; nonfarm refers to income from non-farm activities; offarm refers to income from off-farm activities, agro_natural refers to income from natural resources such as agroforestry, carbon credit, sale of fuelwood etc; livestock_based refers income from rearing of animal and crop_based refers to income from cereals, fruits, and vegetables

To assess the impact of exclosure on livelihood diversification, we identified sixteen different farm, off-farm, and non-farm income activities. On average, households were engaged in just over three livelihood sources with downstream households significantly more diversified than those in open grazing areas (3.24 vs 2.82 sources; Table 4). For analytical clarity, activities were grouped in into six categories: crop-based, livestock-based, off-farm, non-farm, natural resource–based, and government transfers. Crop-based activities were the most common source of livelihood for both groups, with over 96% of households participating in it. Livestock-based activities, including dairy farming, poultry, and beekeeping, represented the second most important source of livelihood. The third and fourth ranked source, however, differed between groups: among households in open grazing areas, government transfers and off farm were the third and fourth most common livelihood sources, whereas among households, the order of these livelihood sources are reversed (Fig. 2). The fifth and sixth ranked livelihood sources—non-farm and agro-natural—were similar for both groups, although the latter contributed more to the livelihoods of the households downstream of an exclosure.

**Table 4:** An independent samples *t* test assessing differences in the mean number of livelihood sources

Group	obs	mean	Std.Err.	Std. Dev.	t	P>|t|	
Open grazing	243	2.82	0.09	1.502	–2.92	0.001	
Exclosure	248	3.24	0.10	1.673			
SDI							
Exclosure	248	0.18	0.005	0.086			
Open grazing	243	0.16	0.006	0.092			

The mean SDI was 0.18 for exclosure areas and 0.16 for open grazing, with a minimum SDI of 0, indicating households with a single livelihood source, while the maximum of 0.6 indicated relatively high diversification (Table 4).

Table 5 presents the factors influencing livelihood diversification. Covariates were checked for multicollinearity using pairwise correlation. Continuous variables such as age, education, distance to farmers’ training centers and market, and livestock numbers were winsorized^7^ to minimize the influence of outliers. Age, gender, land management (exclosure vs. open grazing), extension service, access to credit and marketing information, and livestock numbers were found to affect rural livelihood diversification in Tigray significantly. Land management (exclosure vs. open grazing) was positively associated with livelihood diversification, showing that households living downstream of the exclosure had a significantly higher level of livelihood diversification compared to households utilizing open grazing areas. Specifically, households in open grazing areas were associated with a 0.0211 lower Livelihood Diversification Index relative to households in exclosure areas.

**Table 5:** Determinants of livelihood diversification: results from the fractional logit model

Livelihood index	Dy/dx	Std. Err.	*P* value
Land management (ref=open grazing)	0.0228	0.0074	0.002***
Age of the household head in years	0-.0013	0.0004	0.000***
Gender (ref=male)	–0.0234	0.0113	0.037 **
Education in years	0.0002	0.0013	0.873
Married (ref=unmarried)	0.0129	0.0109	0.233
Family size (no of persons)	–0.0022	0.0018	0.231
Distance to market (walking in minutes)	0.00002	0.0001	0.840
Farm size in hectares	–0.0002	0.0026	0.932
TLU (tropical livestock unit)	0045	0.0019	0.018***
Extension service (ref=no)	0.0240	0.0082	0.003 ***
Access to agricultural inputs (ref=no)	0.0066	0.0076	0.386
Membership organization(ref=no)	0.0002	0.0078	0.979
Agroecology (midland)	0.0345	0.0799	0.66
Agroecology (lowland)	–0.0042	0.0838	0.960
Access to credit	–0.3186	0.0582	0.000****
Access to marketing information	0.2050	0.0583	0.000***

Significant at 10% (*p* < 0.10); ** significant at 5% (*p* < 0.05); *** significant at 1% (*p* < 0.01)

The age of respondents is negatively associated with the LDI and is statistically significant. Holding other factors constant, a one-year increase in the age of the household head is associated with an average decrease of approximately 0.0013 in the LDI. The fractional logit model estimates that female-headed households have a 0.0235 lower LDI compared to male-headed households, indicating the existence of gender disparities in livelihood diversification.

Institutional factors were found to have a significant influence on livelihood diversification. Farm households with access to agricultural extension services had a higher probability of diversifying their livelihoods compared to those without such access. Moreover, the number of livestock units (in TLU) was also significant and positively associated with households’ livelihood diversification. Although livestock ownership constitutes one livelihood source within the diversification index, it also plays a broader intermediary role by supporting crop production through draft power, manure and asset buffering. Consistent with this role, households with higher livestock holdings tended to engage in wider range of additional livelihood activities, suggesting the positive association between asset ownership, diversification and agricultural performance.

Livelihood diversification and agricultural productivity may be correlated. To assess whether exclosure based land management translates into improved agricultural performance, we next examine its impact on crop productivity.

## **Impact of Exclosure on Crop Production**

Average crop yield (kg/ha) was calculated following the method of Wineman et al. (2019), by dividing total standardized crop harvest by cultivated area for each of the production years. Prior to impact estimation, covariate balance between the treatment and control groups was assessed using SMD. Initial imbalances were successfully corrected through matching and entropy balancing, with all SMDs reduced to near zero (Appendix IV, Figs. 6, 7). Furthermore, observations outside the region of common support were removed to ensure credible comparisons.

In the first production year (2023), households downstream of the exclosure achieved notably higher crop yields than those with open grazing areas (Table 6). Estimated ATT effect ranges from 188 to 222 kg/ha, while ATE estimates range from 187 to 251 kg/ha, indicating yield gains of roughly 25-30% relative to open grazing areas. ATU estimates are also positive, indicating that households currently in open grazing areas could achieve similar yield gains if exclosure management were adopted. These consistent results across all matching algorisms suggest the substantial productivity benefits of exclosure.

**Table 6:** The propensity scores matching and the entropy balance model estimation

Year		Effect	PSM	Entropy	NN	Radius	Kernel
		ATT	215.94 (104.63) [0.008] ***	203.92 (81.19) [0.012] ***	222.48 (68.63) [0.001] ***	188.27 (79.8) [0.018]	220.32 (75.55) [0.004] ***
		ATE	187.16 (82.84) [0.024]	204.37 (81.27), [0.012]	250.79 (73.67) [0.000] ***	250.79 (73.67) [0.000]	250.79 (73.67) [0.000] ***
		ATU	247.41 (106.60) [0.020]	225.59 (68.14) [0.001] ***	146.92 (92.82) [0.1134]	207.38 (72.77) [0.0044] ***	209.57 (71.51,) [0.0034] ***
Year 2		ATT	-42.57 (233.36) [0.855]	-215.32 (167.86) [0.200]	-205.59 (147.88) [0.165]	-135.37 (123.79) [0.219]	-105.22 (111.68) [0.220]
		ATE	-71.25 (179.33) [0.691]	-197.46 (167.17) [0.238]	–	–	–
		ATU	100.53 (184.81) [0.586]	–110.01 (154.16) [0.476]	–85.29 (158.38) [0.590]	–106.21 (117.60) [0.366]	–120.36 (117.25) [0.305]

Note, *** are levels of significance. Bootstrapped Standard errors and *P* values are in parentheses and brackets, respectively.

In contrast, no statistically significant yield difference is observed in the second production year (2024/2025). ATT, ATE and ATU estimates are small are small and not significant across all methods (Table 6). This shift may be explained by the relatively favorable rainfall conditions in year two (see appendix II Fig. 4), which appear to have narrowed the productivity gap between exclosure and open grazing lands.

Table 7 presents the effect of the exclosure on crop productivity using the panel data. The HT model estimator is employed to evaluate the impact of the exclosure on crop productivity across two consecutive production years. Variables such as soil fertility, plot slope, seed usage per hectare, labor input per hectare, and fertilizer application were treated as time-varying endogenous variables, while agricultural extension services and the interaction between land management and production years were time-varying exogenous variables. Age, family size, education, and livestock holdings were time-invariant variables, with exclosure status classified as a time-invariant endogenous variable. After an endogeneity test, soil fertility was found to be exogenous and treated as a time-varying exogenous variable. The model showed strong goodness of fit (Wald chi² = 1129, *p* < 0.0001), confirming its suitability for analyzing the effect of exclosures on crop productivity over two years. Our results indicate that households downstream of exclosures produced, on average, 586.6 kg/ha more grain than those in open grazing areas during the baseline year, controlling for difference in soil fertility, labor, fertilizer, and other socioeconomic and institutional factors. This suggests exclosure management provides a substantial productivity advantage, consistent with documented ecological functions of exclosure such as improved soil fertility, enhanced water retention, and groundwater recharge, reported in previous studies in similar environments. The positive impact of exclosures was more pronounced in less favorable years, reflected in a significant negative interaction between land management and year (−298 kg/ha, *p* = 0.035), indicating greater benefits during the first, drier, year. The positive coefficient for production year shows crop productivity increased in the second year for both management types.

**Table 7:** Hausman-Taylor model estimation of crop productivity (kg/ha)

Variables	Delta-method		
	Estimate dy/dx	Std. Err.	Pr > |z|
Land management (ref=open grazing)	586.58	135.588	0.000
Production_year (ref=1)	737.77	113.359	0.000
Soil fertility (medium)	220.28	97.381	0.024
Soil fertility (highly fertile)	105.67	121.915	0.386
Access to extension service (ref=no)	196.60	83.470	0.019
Access to agricultural inputs(ref=no)	–141.91	122.883	0.248
Interaction: land management#year	–297.79	151.247	0.035
Labor per hectare	10.35	1.608	0.000
Total fertilizer (kg)	–20.70	10.185	0.042
Medium slope (ref=flat slope)	40.95	129.213	0.751
Steep slope (ref=flat slope)	–87.87	177.461	0.621
Seed used per hectare kg	–0.28	0.221	0.326
Gender (ref=female)	217.24	107.889	0.044
Midland agroecology(ref=highland)	240.28	104.225	0.021
Lowland agroecology (ref=highland)	119.74	111.217	0.282
Age in years	–3.16	3.178	0.320
Education in years	13.49	12.746	0.290
Membership inorganization(ref=no)	–56.99	81.738	0.486
Access to credit (ref=no)	–113.89	79.644	0.153
Tropical livestock unit	172.65	29.804	0.000
Married (ref=unmarried)	–47.57	112.646	0.673
Access to irrigation (ref=no)	21.73	99.633	0.827
Distance to FTC in walking minutes	3.86	1.633	0.018
Family size (No of persons)	18.69	21.243	0.379
Distance to the nearest feeder road (walking minutes)	–5.55	3.134	0.077

Higher soil fertility and labor input per hectare were strongly and positively associated with yields, emphasizing their role in productivity gains. In contrast, fertilizer use was negatively associated with crop productivity, possibly indicating inefficient application or diminishing returns. Additionally, gender, tropical livestock units, agroecological zones (especially midland), and proximity to farmers’ training centers were positively linked to productivity, while greater distance to feeder roads had a marginally significant negative effect.

The stronger productivity effects observed during the drier production year indicate that exclosure plays an important buffering role under climatic stress, enhancing yield stability rather than merely increasing output in favorable conditions.

## Discussion

Our study reveals that the widely publicized ecological restoration in Tigray is associated with improved household-level socio-economic outcomes. This study generates new empirical evidence on the socioeconomic impacts of exclosure-based restoration by explicitly linking ecological restoration to crop productivity and livelihood diversification using household level panel data. Unlike most prior studies, which primary focused on biophysical indicators such as vegetation cover, soil carbon, and biodiversity, this study quantifies how restoration outcomes translate into measurable economic benefits for rural households. By combining matching techniques with panel econometric models, the analysis advances the restoration-livelihood literature by demonstrating that exclosures provide not only ecological benefits but also climate risk buffering mechanism that help stabilize agricultural production during drought years. We find that exclosure contributes to higher livelihood diversification and crop productivity compared with open grazing lands. These methods address observable heterogeneity, and unobserved time invariant factors strengthening confidence in the estimated associations.

Previous studies have shown that exclosure enhances vegetation cover, soil fertility and water retention and infiltration (Araya et al. 2023; Teku et al. 2025). Our findings extend this literature by demonstrating that these ecological improvements translate into measurable household level economic gains, reflected in more diversified income sources and higher agricultural yields.

Livelihood Diversification scores were significantly higher for households located downstream of the exclosure than for those in open grazing. This suggests that ecological restoration indirectly expands livelihood opportunities by improving resource availability and stability, thereby enabling complementary income activities such as beekeeping, livestock fattening, dairy production, and firewood collection (Abdo and Muluye 2022; Hailemicheal et al. 2024). Similar linkages between ecosystem restoration and diversified livelihoods have been reported in community-managed forests in Uganda and protected areas in Kenya (Mawa et al. 2023; Sun et al. 2023). By providing quantitative evidence based on household panel data, our study goes beyond the qualitative insights of earlier land management research.

The LDI results, however, were low. The computed SDI for both the households living downstream of the exclosure and open grazing areas were below the diversification threshold proposed by Werdofa et al. (2024) and much lower than the pre-war mean SDI of 0.38 reported for Tigray (Gebretsadik et al. 2020). The 2020–2022-armed conflict probably contributed to the decreased values. Structural challenges, such as small landholdings, poor access to markets and credit, weak extension services, and ecological constraints in marginal landscapes, also likely play a significant role. Additionally, although our survey included data from households downstream of the young, mid- and old age exclosures, there remains debate over when and how restored ecosystems begin to generate tangible economic benefits (Zheng et al. 2018). It may take time for the ecological restorations to translate into measurable livelihood outcomes, suggesting a delayed effect. These factors may provide limitations to the interpretation of our results and taken together, they might help explain why SDI values in Tigray remain lower than those observed in other Ethiopian regions (Alemu, 2023; Liyew and Damtie 2024; Taye et al. 2024).

Household demographics and institutional factors further shaped diversification outcomes. We find that age influences the willingness to pursue alternative income opportunities, although this contrasts with studies by Tsegay et al. (2021). Older farmers may remain more strongly attached to crop production, consistent with risk-averse behavior, while younger households demonstrate greater flexibility (Abera et al. 2021; Emeru et al. 2022; Haile et al. 2025). A gender disparity was also observed in livelihood diversification, with female-headed households exhibiting lower levels of diversification. This likely reflects inequalities in access to resources and labor, a pattern consistent with findings from other African contexts (Takane, 2009; Yussuf and Mohamed 2022), although Tsegay et al. (2021) reach the opposite conclusion. Institutional support also mattered: access to extension services and market information was positively correlated with diversification, mirroring results from other regions of Ethiopia (Abeje et al. 2019; Alemu, 2023; Emeru et al. 2022) and other African countries (Ojo et al. 2024; Sato et al. 2025) but differing from the findings from Ethiopia of Guduro Beriso et al. (2022). Interestingly, access to credit was negatively associated with diversification, contrary to expectations and earlier studies from Tigray (Gebretsadik et al. 2020; Gebru et al. 2018), and other regions of Ethiopia (Abera et al. 2021; Emeru et al. 2022; Guduro Beriso et al. 2022; Haile et al. 2025; Taye et al. 2024) likely reflecting the use of credit mainly for subsistence needs or farm inputs, rather than for new ventures, as observed by Tilahun and Holden (2023) and Dinku (2018). Livestock ownership was positively associated with diversification, with animals serving as productive and buffer assets that enable households to branch into trade or non-farm enterprises, consistent with Dereje et al. (2024), although contrasting with pre-war results from eastern Tigray (Gebru et al. 2018).

Crop yield results further illustrate the stabilizing role of exclosure. Using multiple matching methods, we found that during the drought-affected 2023 cropping year, an increased crop yield in the households living downstream of the exclosure was recorded, compared to the open grazing areas (see Appendix I Figure 4). A higher yield advantage was consistently recorded in the households living downstream of the exclosure using the panel data, confirming the robust findings from the PSM. Both models considered land characteristics such as soil fertility, labor, fertilizer, and seed inputs, indicating that the difference in amount of crop yield or the magnitude primary reflects the temporal scope and methodological approach than omitted variables.

In contrast, the yield difference was not statistically significant in 2024, a year with normal-to-above-normal rainfall. This pattern suggests that exclosure functions primary as a risk reducing system, stabilizing production during moisture stress. This interpretation is consistent with earlier biophysical studies documenting improved soil moisture retention, reduced erosion, and increased vegetation cover in exclosures (Araya et al. 2023). In addition, by supplying fodder, fuelwood, and bee forage, exclosure generates indirect livelihood benefits that can support agricultural productivity and resilience (Gebregziabher et al. 2017; Mekuria et al. 2017; Mezgebo et al. 2022).

The crop yield was also shaped by household socioeconomic profiles and institutional factors. Positive correlations found between productivity and soil fertility, animal ownership, and access to extension services highlight the importance of natural and institutional capital. But there were unexpected findings as well. Farmers who lived farther away from training facilities produced higher yields, suggesting that existing training programs might not be effective or sufficiently tailored to farmers’ needs. Male-headed households produced more, likely reflecting culturally defined gender roles: female headed households face labor constraints during peak agricultural periods, as ploughing requires physical strength and usually women tend to spend more time on family care reducing the hours they have. Fertilizer use was negatively associated with productivity, which may suggest that chemical fertilizers and leftover organic fertilizers used during the conflict interact, or that general fertilizer recommendations do not correspond to local soil needs (Abay et al. 2022). Disruptions to fertilizer delivery due to conflict may also have compromised effectiveness.

In summary, our study demonstrates that exclosure, although primarily designed as an ecological restoration intervention, also delivers significant socio-economic benefits by supporting livelihood diversification and enhancing agricultural stability, particularly during drought periods. Communities in these areas experienced both higher crop yields and greater livelihood diversification because of the buffering services provided by restored landscapes. These economic gains, in turn, strengthen incentives for local farmers to manage exclosures sustainably, thereby addressing one of the major drivers of forest degradation in Tigray and across East Africa. The advantage of the crop yield in the exclosure over the open grazing land in 2023 is an indication that exclosure improves farmers’ capabilities to cope with climate shocks such as rainfall variability, which is a persistent challenge to agricultural productivity in the Horn of Africa. Furthermore, these stabilizing effects of the exclosure against the rainfall variability reduce pressure to expand farming into forests and the fragile ecosystems, mitigating risks of land degradation and land use change (Hishe et al. 2021).

## Conclusion

Our study demonstrates that exclosure, as a dryland restoration strategy, provides important ecological and socio-economic benefits for rural communities in Tigray, Ethiopia. Households situated downstream of exclosures recorded higher livelihood diversification and crop yields than those with open grazing lands, indicating that exclosure strengthens household capacities by expanding access to resources, skills, and income opportunities beyond crop and livestock production. Diversification outcomes were influenced by demographic, socioeconomic, and institutional factors, and the positive impact of exclosure was particularly evident under drought conditions, suggesting a role in fostering more resilient farming systems. Overall, while the observed diversification levels remain modest, partly due to the conflict that disrupted livelihood activities, our findings nonetheless confirm that exclosure not only restores degraded ecosystems but also support household resilience through improved productivity and diversification opportunities. These outcomes highlight the importance of scaling up exclosure-based restoration as an integrated land-use strategy in drought-prone, resource-limited environments. However, this depends on supportive demographic, institutional, and structural conditions. Exclosure should therefore be recognized as multifunctional land-use systems that integrate livelihood objectives with conservation goals. Strengthening extension services, improving access to markets and information, and designing gender-sensitive programs would enhance households’ ability to diversify. Likewise, restructuring credit instruments to encourage productive investment rather than short-term consumption is essential. In post-conflict Tigray, rebuilding disrupted non-farm livelihoods should be a priority to ensure that households can fully capitalize on the opportunities created by restored ecosystems.

It is important to note, however, that this study relied on farmers’ self-reported data collection over two years. Long-term research that includes additional factors such as livestock productivity and comparisons with other management approaches, such as irrigation, would provide deeper insights into the sustainability and relative advantages of exclosure., Future research would also benefit from including an assessment of the nutritional and ecological value of crop productivity, and how it differs between exclosure and non-exclosure areas.

## Supplementary information

Appendices

## Data Availability

No datasets were generated or analysed during the current study.

## Footnotes

1 Zoba is an administrative hierarchy in Ethiopia, positioned just below the regional administration.

2 Woreda is an administrative unit that is directly below the zoba administration.

3 Tabia or Kebelle is the administrative unit next to the woreda administration.

4 See the supplementary files for the list and categorisation of areas studied.

5 Village is the smallest administrative unit below the kebele or tabia.

6 See supplementary files for more details.

7 The transformation of statistics by limiting extreme values in the statistical data to reduce the effect of possibly spurious outliers. In this case, the limits were set at the 1st and 99th percentiles.
