# Supplementary material for: Exclosure Enhances Crop Yields and Rural Livelihood Resilience in Northern Ethiopia
Source: Environ Manage. 2026 Mar 31;76(4):132. doi: 10.1007/s00267-026-02413-4 (PMC13038798; doi:10.1007/s00267-026-02413-4)
Supplement: Supplementary file 1 — Appendices [file 267_2026_2413_MOESM1_ESM.docx]

**Appendices**

Table 1: Name of selected exclosure by age, agroecology, number of respondents, etc.

| Name | Age | Agroecology | Exclosure group | Control group | Tabia/kebelle | District |
| --- | --- | --- | --- | --- | --- | --- |
| *Arera* | 33 | Highland | 32 | 31 | *Ayba* | Alaje |
| *Maybeati* | 22 | Highland | 26 | 27 | *Aynbrkeken* | Degua tembien |
| *Zban Birle* | 17 | Highland | 19 | 20 | *Micheal Emba* | Tsirea wenberta |
| *Seyabo* | 33 | Midland | 18 | 19 | *Weyenti* | Adwa |
| *Abel dega* | 22 | Midland | 38 | 38 | *Hayelom* | Tsirea wenberta |
| *merere* | 17 | Midland | 26 | 27 | *Merere* | Kola tembien |
| *Chilko misreta* | 33 | Lowland | 36 | 28 | *Tsigea* | Raya azebo |
| *Tseda Emni* | 22 | Lowland | 24 | 24 | *Nebar Hadnet* | samre |
| *Tunseka* | 17 | Lowland | 29 | 29 | *Koraro* | Hawzien |
| Total |  |  | 248 | 243 |  |  |

Table 4. An independent samples t-test assessing differences in the mean number of livelihood sources

| Group | obs | mean | Std.Err. | Std. Dev. | t | P>\|t\| |  |
| --- | --- | --- | --- | --- | --- | --- | --- |
| Open grazing | 243 | 2.82 | .09 | 1.502 | -2.92 | 0.001 |  |
| Exclosure | 248 | 3.24 | .10 | 1.673 |  |  |  |
| SDI |  |  |  |  |  |  |  |
| Exclosure | 248 | 0.18 | 0.005 | 0.086 |  |  |  |
| Open grazing | 243 | 0.16 | 0.006 | 0.092 |  |  |  |

**Appendix I:** This figure illustrates temporal trends in crop productivity (crop_productivityy0) across three agroecological zones: Highland, Midland, and Lowland. The X-axis represents the year, and the left Y-axis shows crop productivity. Individual observations are displayed as scatter points, with marker shape indicating agroecology (circle = Highland, square = Midland, triangle = Lowland) and color intensity representing precipitation (light = low, dark = high).


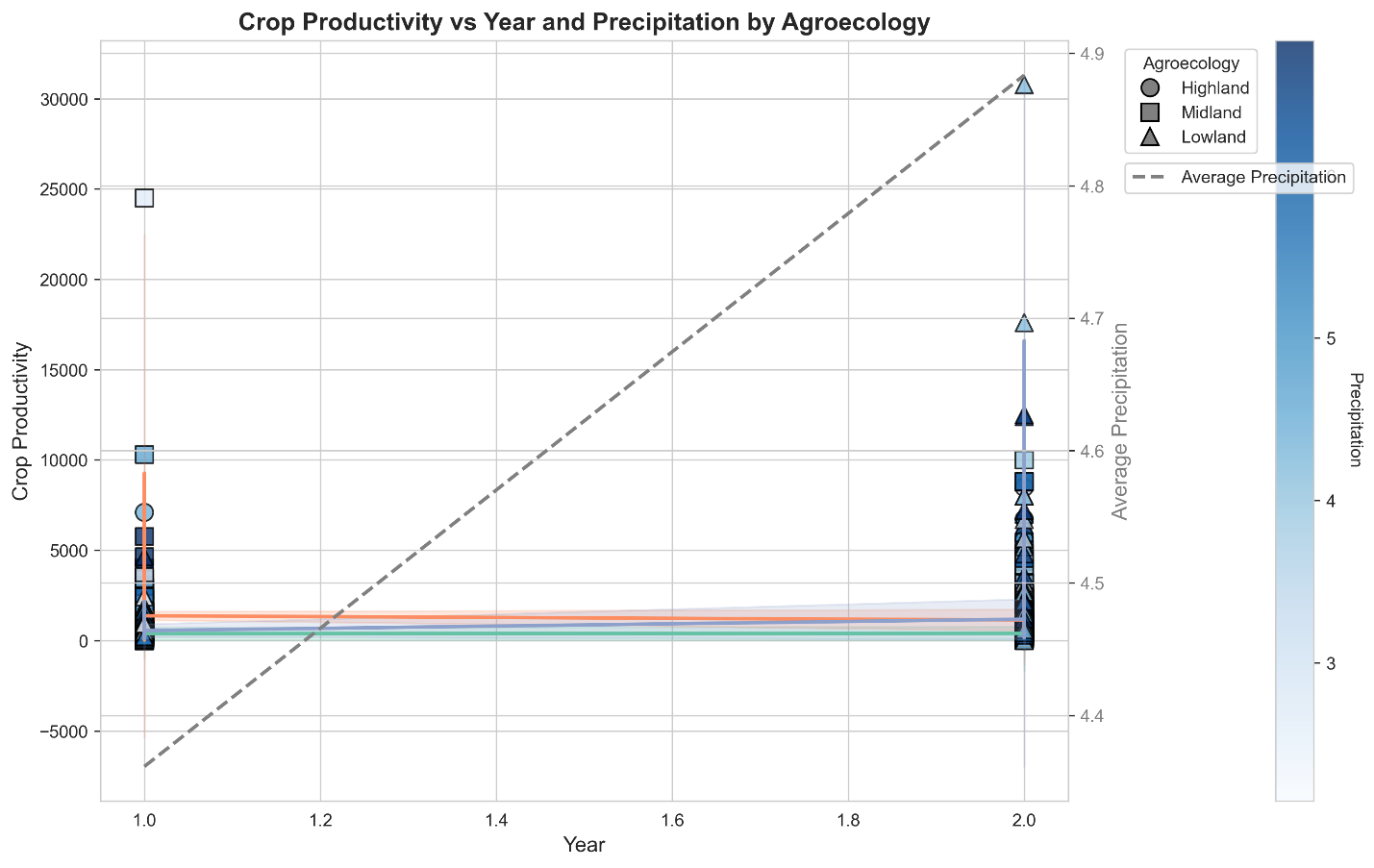


Figure 3: Crop productivity vs year and precipitation by agroecology

**Appendix II.** Daily precipitation trends and seasonal differences (2023–2024). Panel A shows daily precipitation (faint gray) and 7-day rolling averages (colored lines) for nine villages. Panel B shows the percentage change in total precipitation during July–September 2024 relative to 2023: Mokoni (+64%), Koraro (+67%), Nebar Hadnet (+33%), Aynbrkekin (+59%), Weyenti (+46%), Micheal Emba (+136%), Merere (+43%), Ayba (+49.9%). Rainfall was relatively lower in 2023, when crop yields in exclosure-downstream fields exceeded those in open grazing lands, indicating a buffering effect. In 2024, yields were not significantly different, consistent with higher rainfall.


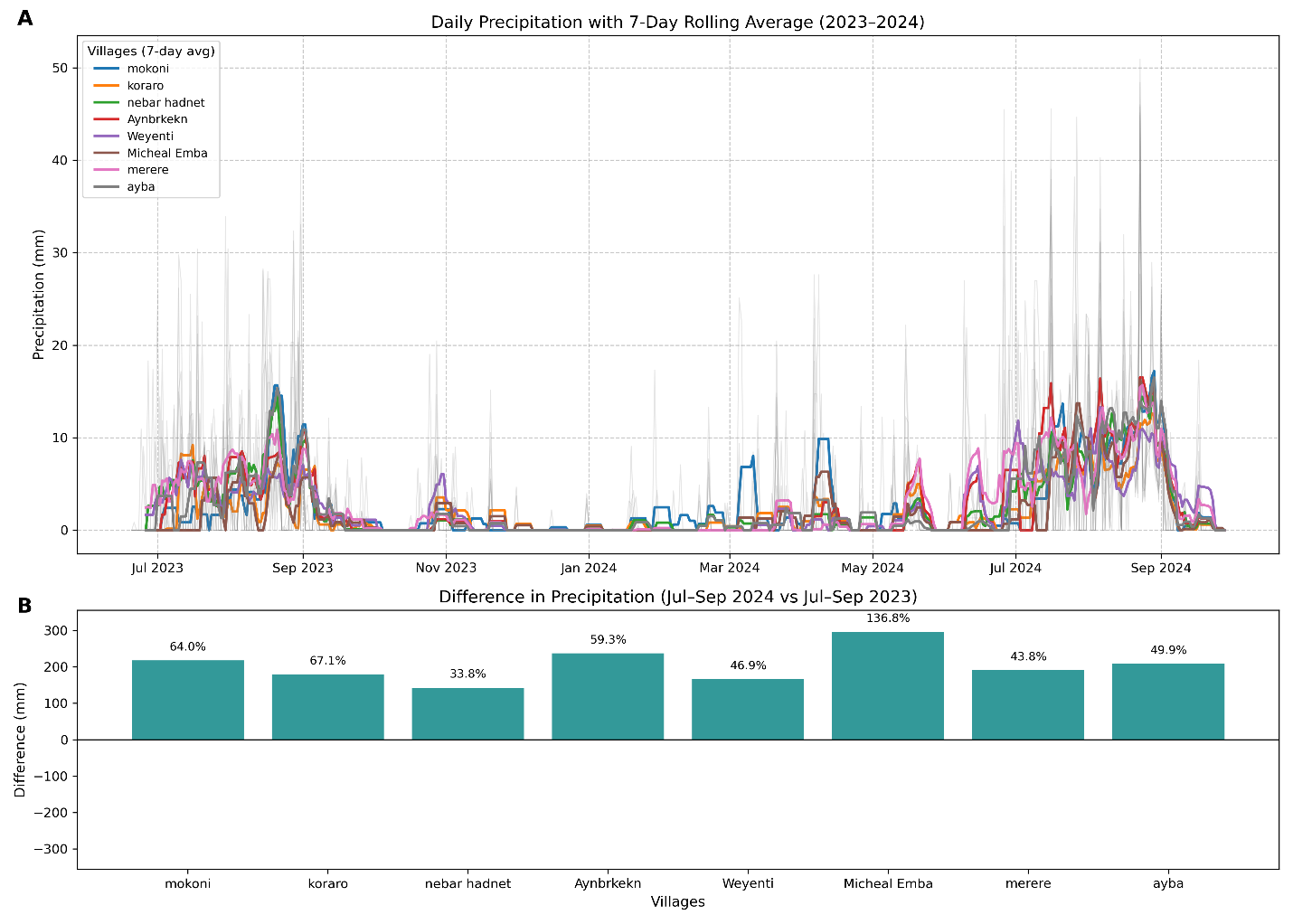


Figure 4: Daily precipitation trends and seasonal differences

Source: Precipitation (CHIRPS)

Appendix III: propensity score vs crop yield

| 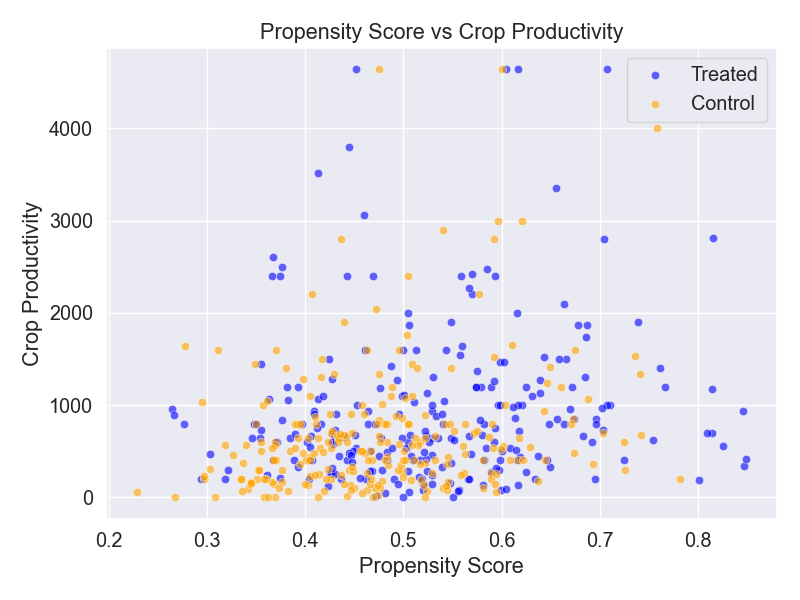 | 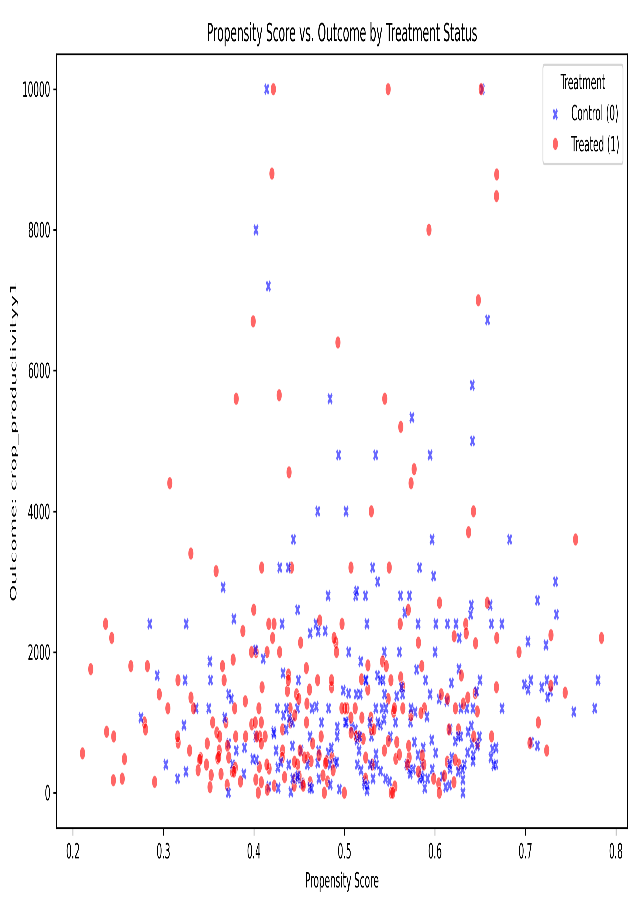 |
| --- | --- |

Figure 5: the propensity score vs outcome variables for year 1 in the left and year 2 in the right by treatment status

**Appendix IV:** The standard mean difference year 1
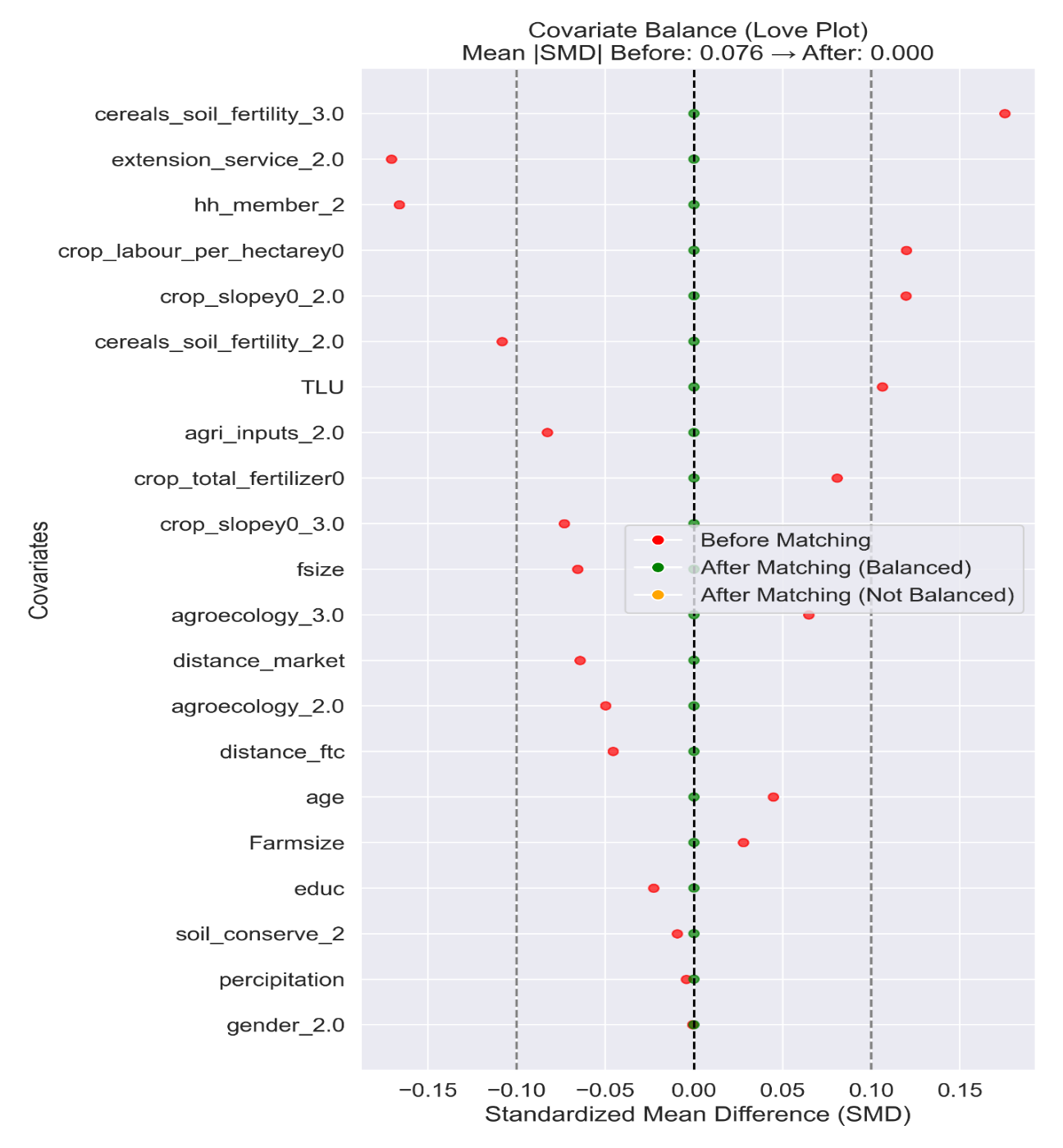


Figure 6: standard mean difference year one data

Figure 6 represents the standardized mean difference of the covariates before matching and after matching. The Y- axis lists the covariates included in the estimation, and the X-axis showed the standardized mean differences (SMD). The red dot represents the SDM before matching and the green dot represents SMD after machining, with SMD becoming almost zero after matching suggesting matching procedure was successful. Trimming was also applied to observations that fell outside the region of common support, which are displayed as gray dots in Figure 6. The covariates included in the estimation were: soil fertility (cereals_soil_fertility_2 and cereals_soil_fertility_3, with cereals_soil_fertility_1 as the reference), extension service (extension_service_2 with extension_service_1 as the reference), household membership in local organizations (hh_member_2 with hh_member_1 as the reference), crop labor per hectare (crop_labor_per_hectare0), farm plot slope (flat-crop_slope_1, moderate-crop_slope_2, and steep-crop_slope_3), tropical livestock units (TLU), agricultural input supply (agro_inputs_1 = yes; agro_inputs_2 = no), fertilizer use (crop_total_fertilizer0), household family size (fsize), agroecology (highland-agroecology_1, midland-agroecology_2, and lowland-agroecology_3), household distance to the market (distance_market), distance to the farmers’ training center (distance_ftc), age of the respondent (age), farm size (farmsize), educational attainment of the household head (educ), gender of the household head (gender), farm-level soil conservation practices (soil_conserve_1 = yes; soil_conserve_2 = no), and rainfall (precipitation).


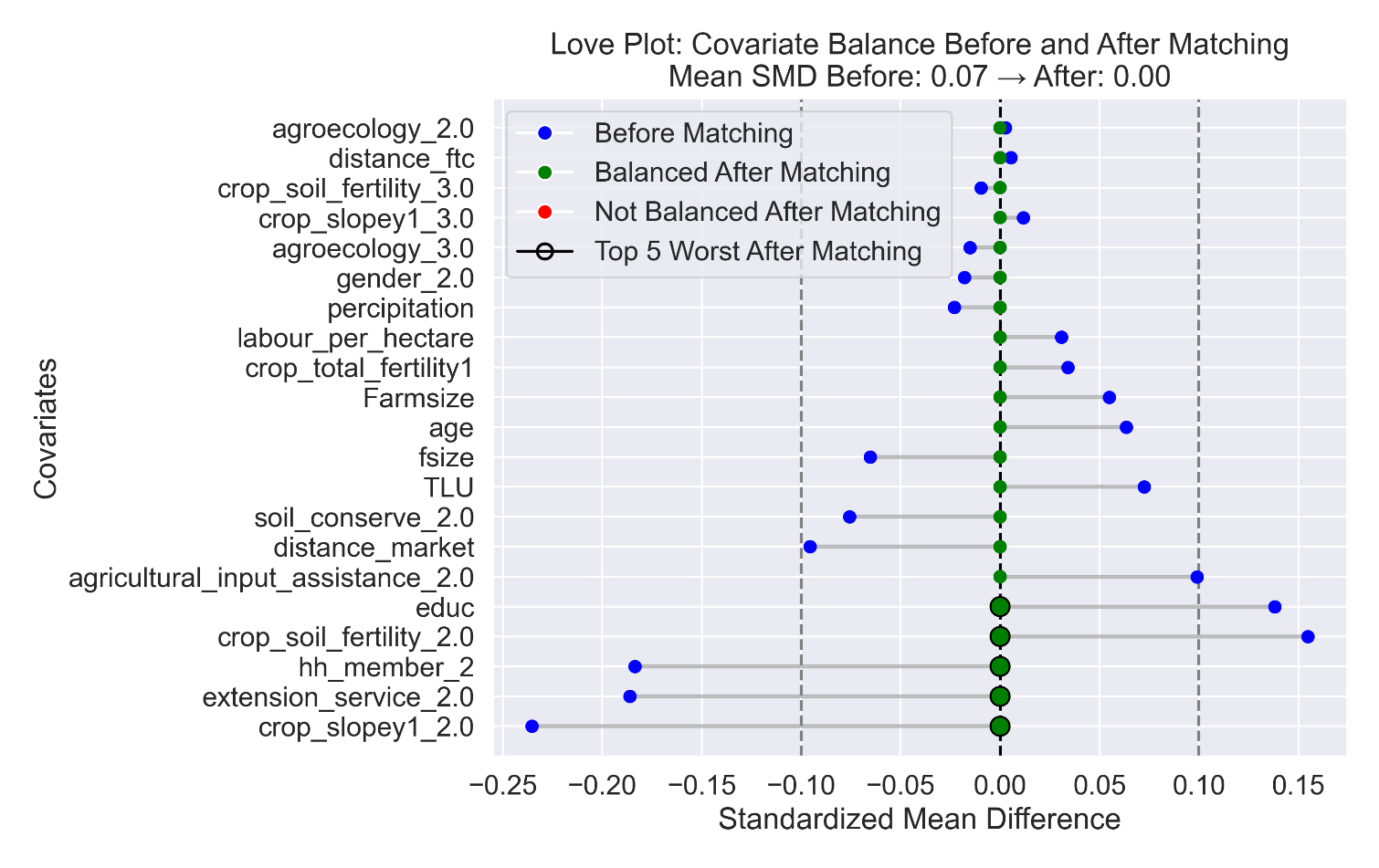


*Figure 7: standard mean difference year 2 data*

Figure 7 represents the standardized mean difference of the covariates before matching and after matching for year 2. The Y- axis lists the covariates included in the estimation, and the X-axis showed the standardized mean differences (SMD). The blue dot represents the SDM before matching and the green dot represents SMD after machining, with SMD becoming almost zero after matching suggesting matching procedure was successful. Trimming was also applied to observations that fell outside the region of common support, which are displayed as red dots in Figure 7. The covariates included in the estimation were: soil fertility (medium - crop_soil_fertility_2 and fertilize- crop_soil_fertility_3, with low- crop_soil_fertility_1 as the reference), extension service (yes-extension_service_2 with no or extension_service_1 as the reference), household membership in local organizations (yes- hh_member_2 withno-hh_member_1 as the reference), crop labor per hectare (labour_per_hectare), farm plot slope (flat-crop_slope1_1, moderate-crop_slope1_2, and steep-crop_slope1_3), tropical livestock units (TLU), agricultural input supply (agro_inputs_1 = yes; agro_inputs_2 = no), fertilizer use (crop_total_fertilizer0), household family size (fsize), agroecology (highland-agroecology_1, midland-agroecology_2, and lowland-agroecology_3), household distance to the market (distance_market), distance to the farmers’ training center (distance_ftc), age of the respondent (age), farm size (farmsize), educational attainment of the household head (educ), gender of the household head (gender), farm-level soil conservation practices (soil_conserve_1 = yes; soil_conserve_2 = no), and rainfall (precipitation).

**Appendix V**: **Bootstrap Distributions of Treatment Effects**

Figure 8 illustrates the bootstrap distributions of the Average Treatment Effect on the Treated (ATT), Average Treatment Effect (ATE), and Average Treatment Effect on the Untreated (ATU) using Nearest Neighbor, Radius, and Kernel propensity score matching. Vertical dashed lines represent mean estimates, shaded regions depict 95% bootstrap confidence intervals, and colors distinguish matching algorithms. The figure demonstrates the magnitude, variability, and statistical significance of exclosure effects on crop productivity across estimation methods, providing robust evidence of the intervention’s impact.


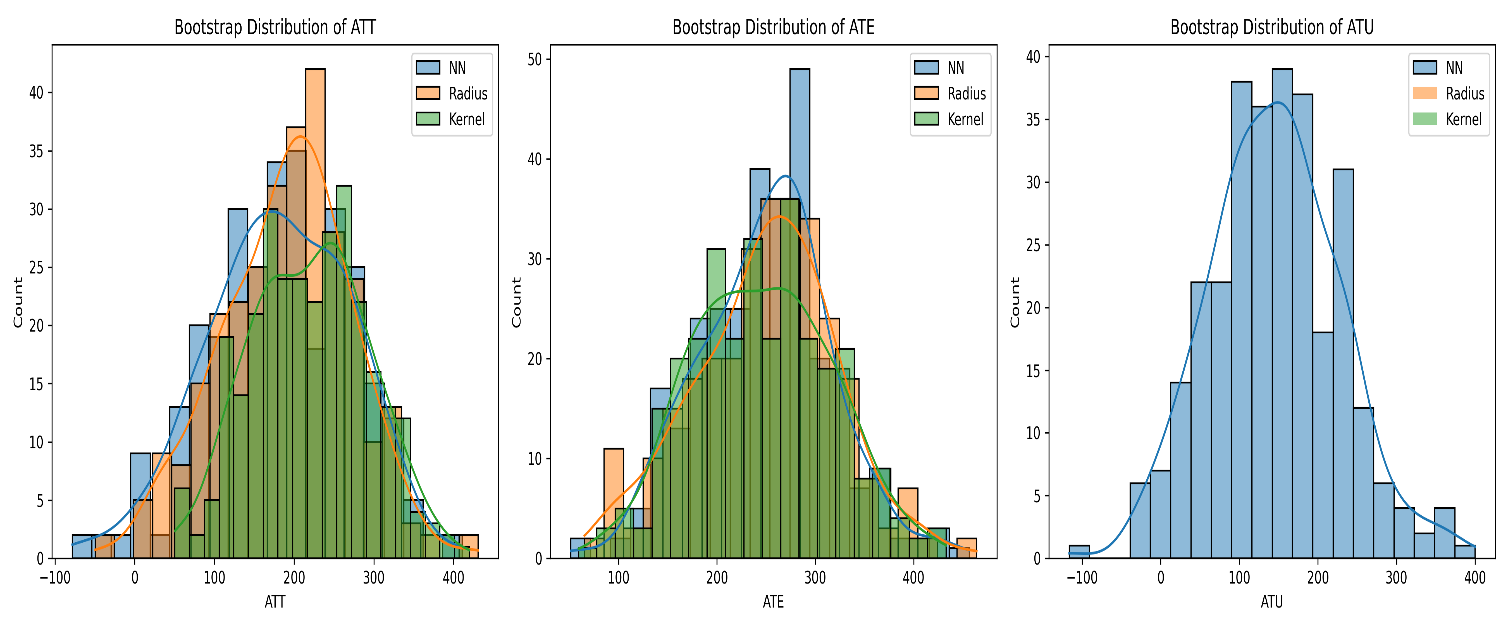


Figure 8: the Bootstrap Distributions of Treatment Effects

**Appendix VI: Post hoc sample size power analysis**

alpha = 0.0500

N = 491

N1 = 250

N2 = 241

N2/N1 = 0.9640

delta = 0.3000

m1 = 0.0000

m2 = 0.3000

sd = 1.0000

Estimated power:

power = 0.9126

To determine whether the sample size was sufficient to show a difference between the two groups (households living downstream of the exclosure and those in open grazing land), we conducted an ex-post statistical power analysis. Using a two-sample comparison framework, with a 5% significance level, the analysis indicates that the final sample 491 households provide approximately 91% statistical power to detect a small to moderate standardized effect size (δ = 0.3). This exceeds the commonly recommended minimum power threshold of 80%*, confirming* that the sample size is adequate for the econometric analysis conducted in this study (see appendix)
